# Feasibility and Effects of an Immersive Virtual Reality Exergame Program on Physical Functions in Institutionalized Older Adults: A Randomized Clinical Trial

**DOI:** 10.3390/s22186742

**Published:** 2022-09-06

**Authors:** Pablo Campo-Prieto, José Mª Cancela-Carral, Gustavo Rodríguez-Fuentes

**Affiliations:** 1 Department of Functional Biology and Health Sciences, Faculty of Physiotherapy, University of Vigo, 36005 Pontevedra, Spain; 2 HealthyFit Research Group, Galicia Sur Health Research Institute (IIS Galicia Sur), Sergas-UVIGO, 36213 Vigo, Spain; 3 Department of Special Didactics, Faculty of Education and Sports Science, University of Vigo, 36005 Pontevedra, Spain

**Keywords:** virtual reality exposure therapy, digital health, older adults, personalized medicine, videogames, exercise, physical functions, rehabilitation, games for health, aged 80 and over

## Abstract

One of the pillars which underpins active aging is found in the performance of physical activity. While adherence to physical activity programs has traditionally been low in older people, immersive virtual reality (IVR) could provide an alternative and complementary training mode. A randomized clinical trial was conducted to explore the feasibility and effects of a 10-week IVR exergame program on physical functions of 24 institutionalized older adults who were allocated to an experimental group (EG *n* = 13; 85.08 ± 8.48 years) and control group (CG *n* = 11; 84.82 ± 8.10 years). The IVR intervention was feasible, with no adverse effects being reported (no Simulator Sickness Questionnaire symptoms; low negative experience scores on the Game Experience Questionnaire < 0.34/4), no dropouts, high adherence, and good post-gaming usability (System Usability Scale > 73.96%). The EG showed significant improvements: Tinetti scores for balance (1.84 ± 1.06; *p* < 0.001), gait (1.00 ± 1.08; *p* < 0.001), total score (2.84 ± 1.67; *p* < 0.001), and handgrip (4.96 ± 4.22; *p* < 0.001) (pre–post assessment). The CG showed significantly worsened compared to the EG: Five times sit-to-stand test, Tinetti scores for balance, gait, and total score, and the Timed Up and Go test total score (post-assessment). The findings show that the IVR intervention is a feasible method to approach a personalized exercise program and an effective way by which to improve physical function in the target population.

## 1. Introduction

Life expectancy is rapidly increasing and is expected to rise in the years to come, thereby giving rise to an aging population. However, a significant proportion of older people may develop frailty, multi-morbidity, and disability, causing a significant impact both on their quality of life and also on health care and social costs [1,2].

Aging is associated with physiological changes and systemic alterations [3]. These may involve a potential decline in sensory, mental, and physical functions, thus leading to a decrease in motor ability, increased obesity, impaired cognition and psychological disorders, which in turn lead to a lower quality of life [4]. The main priority of successful aging management is to enable older people to be healthy, active, and autonomous for as long as possible [2]. Accordingly, functional decline is an important issue that needs to be managed [5].

One of the keys to active aging lies in the practice of physical activity and exercise on a regular basis [2]. These habits contribute to the improvement of physical and functional capacities, can modify degenerative conditions, and increase the quality of life of older people [4,6].

It should be noted that the risk of falls increases progressively when strength and balance levels decrease [7]. Fortunately, neuromuscular exercise has been effective in preventing falls [8]. In addition, a Cochrane review [9] reported that moderate-to-high-level balance training should be included in multicomponent exercises for older people.

Therefore, it is advisable that strength and balance exercises are present in neuromuscular training aimed at reducing falls and, in turn, should include different tasks that progressively increase in difficulty. Moreover, it is possible that the ideal multicomponent exercise addressed to reducing falls is a combination of training in cognitive abilities (attention and decision making), perceptive abilities, neuromuscular performance (trunk and lower and upper limbs), and cardiovascular endurance [10].

A tool that could be useful to collaborate with active aging strategies is virtual reality (VR), where older people could find the motivation to carry out their physical therapies [11].

VR can be divided into three main categories and each category can be ranked by the sense of immersion, or degree of presence it provides. These categories include non-immersive systems (desktop), semi-immersive projection systems (large screen monitor), and fully immersive systems (head-mounted display—HMD—or cave automatic virtual environments) [12,13].

Immersive virtual reality (IVR) allows users to view content in all directions, providing a first-person perspective and a higher level of immersion based on multisensory approaches [14].

Taking into account that the adherence to physical activity programs has traditionally been low in the senior population [15], this immersive technology could provide an alternative and complementary training mode and a good option to overcome older people’s limitations for exercise. Exergames are games based on exercise or that involve the performance of physical tasks in a virtual environment and have been used successfully to improve physical condition [16] or as rehabilitation tool in different pathologies [17,18].

Besides, if this technology can expand resources for physical and functional rehabilitation programs (multisensory stimulation and greater sensorimotor integration), it is logical to think that IVR could help in the development of neuromuscular programs aimed at preserving physical functions and prevent falls in frail older people [19].

Some current systematic reviews have reported that IVR has been a solution used to support psychological rehabilitation (agoraphobia, psychotic disorders, and anxiety) and cognitive rehabilitation (alterations in executive functions). However, the evidence regarding its use in physical and functional rehabilitation of older adults (with and without pathologies) is limited.

For all the above, our hypothesis is based on the fact that an IVR exergaming program could achieve greater benefits than a traditional exercise program since it could achieve more complete and more attractive neuromuscular programs, increasing the levels of motivation and adherence of the participants.

In light of this, the purpose of this study was to conduct a randomized control trial to analyze the effects of an IVR exergaming program on physical function, quality of life, and parameters related to IVR exposure training across a sample of institutionalized older adults.

## 2. Materials and Methods

### 2.1. Study Design

A randomized clinical trial study was conducted at a senior center in Pontevedra, Spain. The study design was registered on clinicaltrials.gov (NCT04336670).

### 2.2. Participants

**Figure 1:**
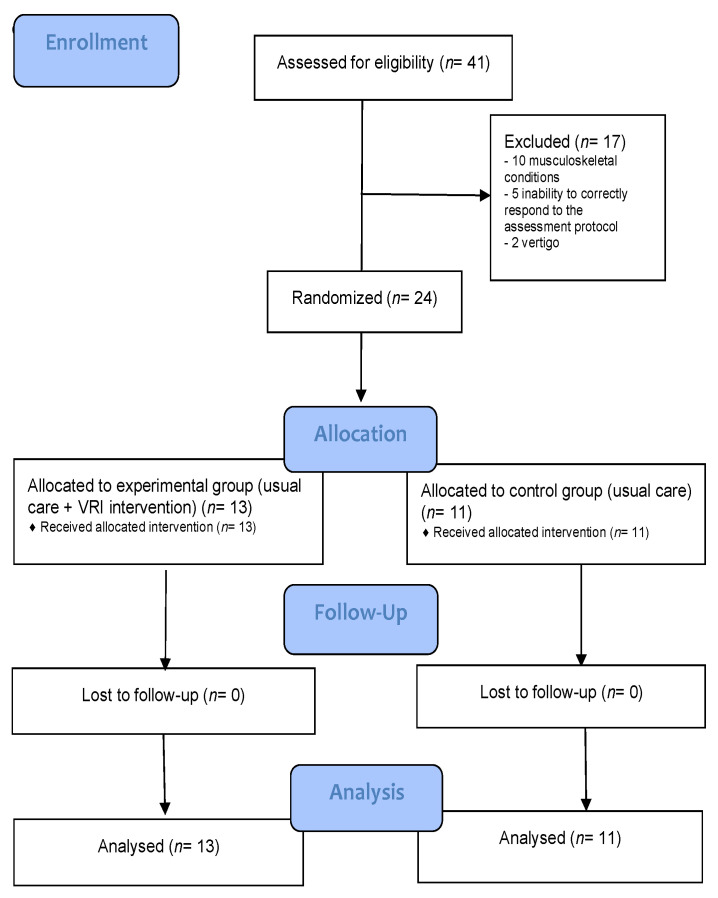
Study design: CONSORT 2010 Flow Diagram.

This study was announced in the senior center chosen. A total of 41 participants applied, all of whom underwent a screening process. All people >65 years old and residents of the senior center were included in the enrollment. Those who had medical conditions that discouraged the practice of physical exercise or who had a physical or cognitive disability that prevented them from standing without aids or understanding the intervention protocol were excluded. In addition, those that presented severe visual impairment, vertigo, epilepsy, or psychosis and that could interfere with the normal performance of the IVR tasks were excluded for caution. Finally, and following the exclusion criteria, 24 out of 41 subjects were recruited. After agreeing to be part of the study, the subjects signed the informed consent and were then randomly assigned to either the control group (CG, *n* = 11) or the experimental group (EG, *n* = 13). Allocation was done by computer. Figure 1 shows this process, and Table 1 summarizes the demographic characteristics of the sample.

The study was undertaken with the understanding and written consent of each participant, with approval from the Servizo Galego de Saúde (SERGAS) Ethics Committee (Ref. 2020/078), and in compliance with national legislation and the Declaration of Helsinki. Participants were required to agree to all aspects of the study and were able to leave the study at any time.

### 2.3. Pre-Intervention

Three weeks prior to the intervention, the IVR equipment was installed in the center’s gymnasium room and training sessions were scheduled for the center’s staff in charge of supervising the sessions. In addition, they were taught how to troubleshoot minor technical issues that might arise with the software and hardware. During this time, therapists and center users could experience various games (mountain landscape or seabed scenarios), and receive instructions on handling. The most commonly used environments were *TheBlue* and *Steam VR Home*, whose features are described below:

*TheBlue*: This is a game based on the observation of the seabed. There are three possible scenarios: one located in a coral reef, another in a sunken ship, and the last one in the abyssal depths of the ocean. This last scenario was discarded because it was considered a claustrophobic and unsuitable scenario. In the coral reef setting, participants could observe and interact with fish, anemones, and jellyfish. In the shipwreck scenario, they could also interact with small fish, and observe manta rays and a large whale. User feedback on these scenarios was very positive.

*Steam VR Home*: This portrays an environment located in a house in the mountains. It allows greater freedom of movement than in the previous game and a greater degree and variety of options in the tasks to be undertaken (painting, designing the room, etc.). It is a very pleasant setting. In particular, the participants very much enjoyed the possibility of butterflies landing on their hands, or simply observing the mountain views on a clear day.

**Figure 2:**
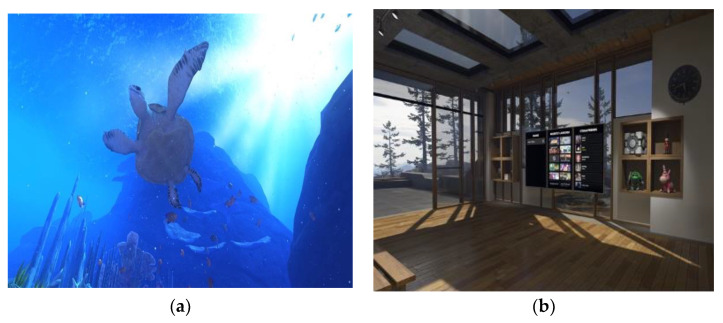
Screenshots of some virtual scenarios proposed for first contact with IVR: (**a**) Seabed and turtles in *TheBlue* experience; (**b**) inside the mountain house in *Steam VR Home*.

In order to verify the presence and immersion in both experiences, the staff in charge of supervising the VR sessions was who guided, proposed different actions to carry out in the virtual experiences, and asked various questions about the virtual environment (i.e., describe a room and its objects, tell the time on the clock in the house, or make changes to the decoration of the virtual scenario). At all times they were warned that they should stop the session if they experienced discomfort or dizziness, although none occurred, and the scenarios were mostly described as pleasant and calm (Figure 2). These tests helped the participants lose their fear of something new and show willingness to use it again.

### 2.4. IVR Intervention

During the study period and the previous acclimatization sessions, approximately 450 IVR sessions were conducted. A total of 30 IVR sessions of an exergame over a period of 10 weeks was programmed for each participant in the EG. Three sessions were held every week and each IVR training session lasted for 6 min. At the same time, both the CG and the EG participated in the usual care programs of the center (including occupational therapy and memory workshops). Some sessions were rescheduled if justifiable absences occurred due to illness, doctor’s appointments, etc.

The place chosen to conduct this study was the center´s gym room (8 m^2^ play areas were delimited within this room). The hardware selected was: HTC Vive Pro^TM^, consisting of an HMD equipped with a wireless adapter, two base stations, and two controllers; desktop computer (CPU: Intel Core I7 7700 at 3.6 GHz, 1 TB HDD Sata 3.5 and NVIDIA GeForce RTX 2070 GPUs); and a LED monitor. Support software was obtained from Viveport (https://viveport.com, accessed on 10 January 2022).

For the choice of the exergame, we looked for an immersive experience that was close to the general exercise recommendations for older people [15]. Starting from the software used in our recent research [20,21], and with the endorsement of four physiotherapists with more than 10 years of experience in the elderly, it was decided to use the *BOX VR* game (available in the library of Viveport.com, accessed on 10 January 2022).

*BOX VR* is able to place the user in a virtual gym with a very large training area and allows them to choose up to four different environments. The challenge consists of achieving a quick motor response to different stimuli based on boxing practice. During the game, the participant stands with one foot in front of the other, and different colored balls are presented to be hit with one or the other hand. Some of them incorporate an arrow that guides the movement to be performed (i.e., the up arrow will be an uppercut, the side arrow will be a cross or a hook depending on the side). In addition to the balls, other stimuli are presented: a diamond that involves blocking with both hands and lateral and/or perpendicular bars to the ground with the intention of moving the trunk, head and lower limbs to vary their position. This game also offers the possibility of working on another important component, such as hand-eye coordination or cardiovascular training.

At the same time, the game is also useful as cognitive stimulation since the random appearance of unexpected stimuli further aids the participants´ work by adding attentional mechanisms, improving concentration on the task and providing greater sensorimotor integration [22].

**Figure 3:**
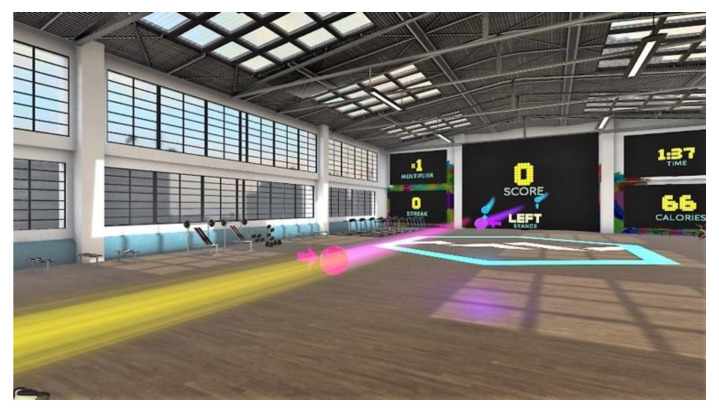
Screenshot of the exergaming proposed (*BOX VR*) placed in a gym.

As mentioned above, the proposed exergame is based on the continuous movement of the body, focused on improving joint ranges, strength, endurance, and muscle power. These objectives are a fundamental part of conventional physiotherapy sessions in older people [23]. In addition, in previous research [24,25], we have shown that this topic (immersive scenarios without accelerations or without sudden changes in point of view) has a low probability of causing cybersickness symptoms (Figure 3).

**Figure 4:**
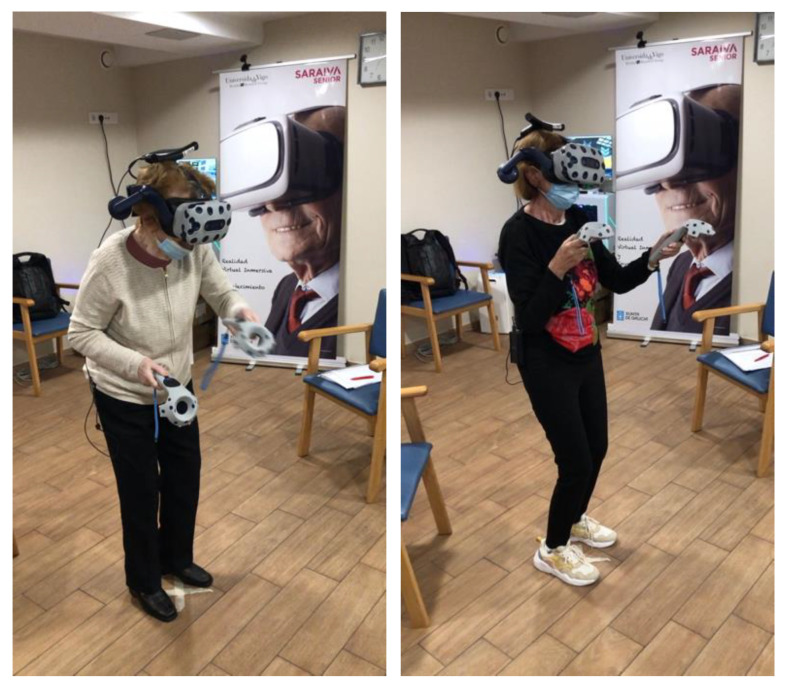
Participants during an exergaming session guided and supervised by a physiotherapist.

Even so, each participant received individual guided and supervised sessions (Figure 4). Three training groups were established for optimal organization, and all the subjects of each group (3–4 participants) were present in the gym at the same time, watching their peers train.

### 2.5. Assessments

Demographic characteristics were collected by trained researchers before the intervention. The experimental protocol involved a set of pre/post- and follow-up evaluations (baseline, 10 and 14 weeks, respectively). Furthermore, one set of extra evaluations was carried out four weeks after the initial evaluation to ensure VR exposure adequacy (intermediate assessment only included parameters related to IVR exposure training). The following parameters were collected in three blocks (physical functions, quality of life, and parameters related to IVR exposure training).

- Physical function was assessed by balance and gait, mobility, lower limb function and handgrip strength. The balance and gait were evaluated by the Tinetti test [26]. Functional mobility and lower limb function were measured by the Timed Up and Go (TUG) test [27] and the Five times sit-to-stand test (FTSTS) [28]. Handgrip strength (HGS) was measured with an analog hand dynamometer. To perform the measurement, the participants in the stand position held the dynamometer with their dominant hand and performed the test with their elbows bent at 90 degrees. These data were collected in both groups in the pre-, post-, and follow-up evaluations.
- Quality of life (QoL) was assessed by the 12-Item Short Form Survey (SF-12), Spanish version 2 [29]. These data were collected in both groups in the pre- and post-evaluations;
- Parameters related to IVR exposure training were assessed for cybersickness, usability, post-gaming experiences, and VR sessions. Cybersickness was evaluated by the Simulator Sickness Questionnaire (SSQ), translated into and adapted for Spanish [30,31,32], usability was measured by the System Usability Scale (SUS) [33], post-gaming experiences were evaluated by Game Experience Questionnaire (GEQ-post game module) [34] and an ad hoc satisfaction questionnaire. Its development is based on the information extracted in our two published systematic reviews [35,36] It has been used in the evaluation of our previous research experiences [20,21,24,25] and was intended to assess the satisfaction of using exercise based IVR in the elderly population. Finally, the VR sessions were evaluated by the number of *BOX VR* sessions completed and their scores. These data were collected in the EG in intermediate and post evaluations.

In addition to these assessment, two extra safety control measures were established: Administration of the SSQ at the end of the first 6 sessions to check the tolerability of the proposed exergame and heart rate monitoring and perceived exertion (measured using the Borg perceived effort scale [37]) throughout the study to detect possible physical overexertion.

### 2.6. Statistical Analysis

Descriptive statistics (mean, standard deviation) were used to represent the demographic characteristics of the sample. For the normal distribution analysis, the sample Shapiro–Wilk test (*p* > 0.05) was used. As the outcomes were not normally distributed, non-parametric tests (Kruskal–Wallis test; intergroup) were conducted to assess the effect of the program between the groups (EG, CG) and in the three moments (pre, post, follow-up). To assess the differences between the two groups (EG and GG) on the FTSTS, Tinetti test, HGS, TUG, and SF-12 over time (post–pre and post–follow), we conducted a multivariate analysis of variance (MANOVA), with “group” as the between-subject factor.

MANOVA assesses multiple dependent variables simultaneously, which can cause the results to be ambiguous at times. When applying MANOVA we cannot say with certainty whether or not it was really the independent variable, or the multiple dependent variables that generated the effects on the dependent variable under study. Another disadvantage of applying MANOVA could be that a lot of data are needed to achieve meaningful results.

We performed all the tests using the Statistical Package for the Social Sciences (SPSS Inc., Chicago, IL, USA) for MAC version 26.0. The level of significance was set at <0.05.

## 3. Results

The IVR intervention was feasible, with no adverse effects being reported (across 100% of the sample), no dropouts, and a high adherence (92.30% of the sample completed all the sessions). Only one participant did not complete all the sessions, and his absence for two weeks had just cause (absence due to quarantine for COVID-19 infection and close contact). No significant differences were observed at the baseline between the groups (sociodemographic data and physical functional tests total scores).

The main outcomes are summarized in Table 2, Table 3, Table 4, Table 5, Table 6 and Table 7. The intergroup and intragroup differences are shown in Table 2 and Table 3.

The CG shows significantly worsened compared to the EG: FTSTS test, Tinetti test scores for balance, gait, and total score, and TUG test total score (post assessment) and Tinetti test scores for balance, gait, and total score (follow-up assessment). At the same time, the EG showed significant improvements in Tinetti scores for balance (1.84 ± 1.06; *p* < 0.001), gait (1.00 ± 1.08; *p* < 0.001), total score (2.84 ± 1.67; *p* < 0.001) and handgrip (4.96 ± 4.22; *p* < 0.001) (post–pre assessment).

Regarding the QoL scores (Table 4 and Table 5), both groups maintained or improved their scores, particularly the mental score. However, the EG obtained significantly improved scores in the physical component, as compared to the CG (post–pre-assessment).

Related to the post-gaming experiences in the EG, Table 6 reports the results after the IVR program in intermediate-post-assessment: no cybersickness symptoms and good scores in usability (>73%). The post-gaming experiences showed low scores for negative experiences (range 0.15 ± 0.29–0.37 ± 0.43/4) and the satisfaction questionnaire (Table 7) highlighted good or very good experiences (100%), recommendations of experience (81.82% of the sample), and the usefulness of the program for peers (81.82% of the sample).

## 4. Discussion

Our study examined the effects of an IVR exergaming program on physical functions, QoL, and parameters related to IVR exposure training, in a sample of institutionalized older adults. The findings show that the IVR intervention is a feasible method to approach a personalized exercise program and an effective way by which to improve physical function in the target population.

Over the past decade, technology-based exercise interventions have gained immense interest worldwide. Specifically, the use of VR as a physical therapy tool in elderly people was the subject of analysis in a systematic review published by our research group recently [35]. In it, some of our general conclusions pointed to the fact that the use of these tools focused on the treatment of older people was still perceived as far away and, although it seems a promising option for the future, more studies aimed at exploring the possible benefits on the physical and functional capacities in senior populations were needed.

In our trial, we implemented a 10-week intervention that was proven to be feasible, with neither adverse effects (without SSQ symptoms) nor dropouts associated with the proposed program. The high number of successful sessions carried out backs up our proposal, with high adherence (12/13 completed all the sessions). This success was made possible by being systematic and rigorous, and following the constraints outlined by Davis et al. [38], which in the past have kept these groups away from IVR technologies: adequate sample selection, state-of-the-art devices, and a suitable virtual experience. Our clinical and research experience in the field of exercise therapy in the elderly [39] and our previous research experience with different exergames [20,24] all contributed to the successful implementation of the program.

Furthermore, IVR provides an artificial interactive environment closely representing reality. Older adults with different conditions can experience varying sensory stimulation in a comfortable and safe virtually simulated environment, which may lead to a boost in motivation and adherence rates to physical exercise programs [39].

The chosen game was safe (no symptoms of cybersickness) and makes significant demands on the physical capacities of the participants, ensuring a multicomponent workout (aerobic capacity, strength, and endurance combined with coordination, agility, and balance training). At the same time, it also achieves cognitive stimulation, since it requires attentional strategies to respond to unexpected stimuli that require different motor responses. We used the multitasking paradigm as an element for cognitive training.

Other interventions with IVR that have sought to improve cognitive aspects have succeeded in improving physical aspects in elderly people with mild cognitive impairment [40,41,42], so it seems appropriate that exercise programs aimed at this population should integrate work on physical and cognitive domains to achieve comprehensive results.

It should be stressed that IVR has several requirements for motor and cognitive rehabilitation interventions: repetitive practice, feedback about performance, multimodal stimulation, and controlled, safe, and ecologically valid environments [43].

Similarly, recent studies have shown that the combination of physical and cognitive factors is the key to improving response times [44], a very important aspect in frail groups and one, according to some authors, whose deterioration could be related to a greater risk of suffering falls in groups such as those affected by Parkinson’s disease [45,46]. Therefore, in future research with IVR, it would be interesting to integrate all of these variables, objectifying physical, cognitive, and response time results in the elderly.

Regarding the effects of our intervention on physical function, the incorporation of the exergaming program to the usual therapies of the center has enabled the EG to avoid the significantly lower values that were recorded by the CG for FTSTS, Tinetti (balance, walk, and total scores) and TUG test (total times). The HGS scores were also lower, although not significantly so. Furthermore, in the intragroup comparison, the EG achieved improvements in FTSTS times and significant improvements in Tinetti (balance, gait, and total scores) and HGS. For the TUG test times, the results were slightly worse (−1.06 ± 4.23 total times), although to a lesser extent than the loss shown in the CG for the same variable (−3.03 ± 4.62 total times). It is possible that the discrete results obtained by the CG in the TUG test are related to the fact that the tasks in the proposed exergaming differ significantly from activities such as “turning” or “stand to sit” (items where there was a greater increase in TUG times).

Nevertheless, from our point of view, the maintenance of functional capacities in this population (aged, 80 and over) can be considered a good indicator, especially when compared with the results obtained in this same variable by the CG.

These results are in line with other studies in which exercise programs undertaken with IVR have improved aspects related to balance [19,41].

As expected, the follow-up did not show statistically significant intragroup differences since both groups continued to maintain the usual therapies carried out in the center. Nevertheless, in general, it could be said that the physical functions between both groups were equal, maintaining only significant intergroup differences for the Tinetti test scores.

Regarding QoL, the CG obtained significant improvements when compared to the EG in the physical score. However, both groups improved their mental scores. These results coincide with those reported in previous studies [14], although the scale used was different, with the physical domains not registering significant values in the VR group. In the review by Cacciata et al. [47], they did not find a robust relationship between exergames and improvement in the QoL of the elderly either. In our case, the improvements experienced in the CG could be due to the activities that were usually carried out in the center, while the improvements of both groups in the mental score reinforce what was previously stated with respect to multidisciplinary evaluations. This suggests that assessing levels of anxiety, depression, and stress in the elderly may be necessary in order to obtain valuable information regarding their QoL after the application of IVR exercise programs.

The results shown in the domain related to IVR exposure were satisfactory. First, the experience was feasible and safe. In line with previous research [21,25], the experience and equipment selected did not cause cybersickness (without SSQ symptoms) in any of the participants in 100% of the IVR sessions. Being a sensitive group, it is very important to use virtual experiences without strong accelerations or abrupt changes in viewpoint.

Secondly, usability was considered good in the two controls carried out (intermediate and final) with values that exceeded 73/100 points. These results are also congruent with other studies that have used this device with similar objectives and in a senior population [14,21,48,49].

Third, post-gaming experiences and satisfaction corroborated the appropriateness of the selected task. The GEQ post-gaming values were residual in the items rating negative experiences (range 0.15–0.37/4), and the satisfaction questionnaire showed that the whole sample considered the experience good (100%), that there was nothing they did not like (72.73%), that they would recommend the experience (81.82%), and also that they considered it useful for people their age (81.82%)—generally because it encouraged them to do exercise. These results are in line with other studies [21,24,50,51,52], although in our case, and in a 10-week intervention, the results have more ecological value than in the analysis of only a few sessions.

In our trial, the participants showed a high level of tolerability to the proposed virtual program. We have successfully incorporated cognitive strategies combined with physical exercise to achieve comprehensive training proposals aimed at older people.

Relating to the overall post-gaming experience, the participants highlighted how fun the sessions were, with a good musical rhythm that helped them to carry out the tasks and a very spacious and comfortable training area (much larger than the usual gym room). In particular, the participants showed very positive opinions linked to the physical exercise performed and the benefits for people like them, as can be seen in the responses to question, *Do you think this could be useful for people of your age? Why?* in Table 7. From our point of view, it was also positive that the sessions (although individual) were witnessed by four other peers who gave positive feedback on the performances of each participant (sometimes even with applause in the room, and at other times the peer group even encouraged participants to attend a session on a day when their mood was not the best).

These findings show that bodily movement and individual sessions are undertaken in a group mode are an engaging factor for older adults, and one which can motivate them to prolong exercise training.

### Limitations

Our study has several limitations: (1) The sample size limits the ability to generalize our results. It is possible that the pandemic situation meant that the initial number of subjects likely to participate was lower than expected due to fear of COVID-19. (2) Possible drawbacks of the chosen statistical analysis technique could influence the interpretation of the results. (3) We compared the EG with the CG where both received a common intervention (the usual center therapies). Therefore, with the methodology used, it is not possible to know with certainty which of the improvements shown are attributable to the IVR program (which ones are to the exercise program or which ones are to the VR exposure) and which to the influence of the usual center therapies. More research is needed where these groups are compared. (4) Another limitation could be the duration of the program. It is possible that establishing a similarity with medium- and long-term benefits that exercise programs achieve in older people, a longer intervention could achieve better results in physical and functional domains and could have influenced our TUG test scores. (5) Following our previous findings, we set the difficulty of the game at an intermediate level. Although the profiles were quite homogeneous, it is possible that in some participants this level was below their actual abilities and as a result they could have experienced less intense and challenging training. If this is so, it is likely to result in fewer training benefits. Since this could have an impact on the benefits of training physical and functional abilities, future research should explore how the level of challenge in the VR game could be tailored to the capacity of each participant without diminishing their sense of autonomy.

## 5. Conclusions

In summary, outcomes show that our 10-week IVR exercise program was feasible and had positive effects on physical functionality in institutionalized older adults, particularly in aspects related to gait, balance, and handgrip strength. Although cognitive function and mood were not direct objectives of this study, the physical and cognitive stimulation of the selected exergame, the results of the mental dimension of quality of life and the satisfaction responses obtained all support the idea that future studies with IVR exercise programs may be considered as a multidisciplinary evaluation offering the holistic assessment of the health of institutionalized older adults.

## Figures and Tables

**Table 1:** Demographic characteristics of the participants.

		Experimental Group (*n* = 13)	Control Group (*n* = 11)
Age (Years)		85.08 ± 8.48	84.82 ± 8.10
Sex	Female (%)	84.61	90.90
Height (m)		1.55 ± 0.09	1.49 ± 0.08
Weight (Kg)		60.32 ± 10.53	61.60 ± 11.75
BMI (Kg/m^2^)		25.25 ± 5.13	27.86 ± 4.09

Mean ± Sd; BMI: Body Mass Index; Kg: Kilograms; m: meters.

**Table 2:** Intergroup differences at the 3 evaluation moments (physical and functional abilities).

		Pre		Post		Follow Up	
		EG (*n* = 13)	CG (*n* = 11)	EG (*n* = 13)	CG (*n* = 11)	EG (*n* = 13)	CG (*n* = 11)
FTSTS	5 Sit to Stand (s)	15.56 ± 4.52	21.19 ± 12.63	13.81 ± 3.46	25.57 ± 14.15 *	17.16 ± 3.88	21.67 ± 10.70
TINETTI	Balance	12.38 ± 1.26	12.27 ± 1.95	14.23 ± 1.09	11.45 ± 1.96 *	13.42 ± 1.44	12.00 ± 1.83 *
	Gait	10.08 ± 1.50	9.09 ± 2.02	11.07 ± 0.86	9.09 ± 1.57 *	10.50 ± 1.17	9.10 ± 1.73 *
	TOTAL	22.46 ± 2.15	21.36 ± 3.59	25.30 ± 1.65	20.54 ± 3.35 *	23.92 ± 2.35	21.10 ± 3.38 *
HGS	Handgrip (kg)	15.54 ± 7.13	15.00 ± 6.35	20.50 ± 6.18	16.95 ± 5.97	21.08 ± 4.98	16.70 ± 7.09
TUG	Sit to stand (s)	2.80 ± 1.20	4.87 ± 2.20 *	3.16 ± 1.88	5.02 ± 3.97	2.64 ± 1.59	3.79 ± 1.93
	Gait (s)	4.94 ± 2.51	5.69 ± 3.31	4.77 ± 0.92	6.49 ± 3.30 *	5.44 ± 3.71	6.45 ± 3.05
	Turning (s)	1.73 ± 0.53	2.46 ± 0.87 *	2.31 ± 1.05	3.61 ± 3.88	1.88 ± 1.80	3.02 ± 1.34
	Return Gait (s)	4.57 ± 2.63	5.51 ± 2.82	4.87 ± 2.62	5.85 ± 2.17	4.88 ± 3.49	5.56 ± 3.20
	Stand_to_sit (s)	3.90 ± 1.01	4.71 ± 2.14	4.51 ± 1.79	5.02 ± 2.71	4.04 ± 1.13	6.54 ± 3.68 *
	TOTAL (s)	17.93 ± 6.37	23.23 ± 9.25	19.00 ± 6.58	26.27 ± 11.77 *	18.88 ± 9.12	25.36 ± 11.01

* *p* < 0.005; Mean ± Sd; CG: Control group; EC: Experimental group; FTSTS: Five times sit to stand test; HGS: Handgrip strength; Kg: Kilograms; TUG: Timed Up and Go test; s: Seconds.

**Table 3:** Intragroup and multivariant differences between moments (physical and functional abilities).

		Differences between Moments (Post-Pre)			Differences between Moments (Post-Follow)		
		EG (*n* = 13)	CG (*n* = 11)	Manova 2 × 2	EG (*n* = 13)	CG (*n* = 11)	Manova 2 × 2
		Dif. Post-Pre	Dif. Post-Pre		Dif. Post-Follow	Dif. Post-Follow	
FTSTS	5 Sit to Stand (s)	1.75 ± 3.63	−4.38 ± 7.44 **	F = 0.544 Sig = 0.465	−3.96 ± 3.87	3.50 ± 9.53	F = 0.016 Sig = 0.901
TINETTI	Balance	1.84 ± 1.06 **	−0.81 ± 1.53	F = 2.064 Sig = 0.048	−0.75 ± 0.86	0.40 ± 1.17	F = 0.503 Sig = 0.482
	Gait	1.00 ± 1.08 **	0.01 ± 0.89	F = 0.125 Sig = 0.725	0.58 ± 0.66	0.30 ± 1.05	F = 0.462 Sig = 0.500
	TOTAL	2.84 ± 1.67 **	−0.81 ± 1.99	F = 0.397 Sig = 0.532	−1.33 ± 1.30	0.10 ± 1.66	F = 0.534 Sig = 0.469
HGS	Handgrip (kg)	4.96 ± 4.22 **	1.95 ± 2.91	F = 0.161 Sig = 0.691	0.33 ± 2.17	−0.55 ± 2.43	F = 0.930 Sig = 0.340
TUG	Sit to stand (s)	−0.36 ± 1.31	−0.15 ± 3.04	F = 3.571 Sig = 0.045	0.46 ± 0.74	0.44 ± 2.45	F = 0.048 Sig = 0.828
	Gait (s)	0.16 ± 2.52	−0.79 ± 4.05	F = 0.248 Sig = 0.621	−0.67 ± 3.55	0.43 ± 1.75	F = 0.461 Sig = 0.501
	Turning (s)	−0.58 ± 1.27	−1.15 ± 3.48	F = 0.340 Sig = 0.563	0.47 ± 2.05	0.50 ± 1.45	F = 1.349 Sig = 0.252
	Return Gait (s)	−0.29 ± 1.63	−0.33 ± 1.71	F = 0.928 Sig = 0.341	−0.08 ± 2.08	0.072 ± 1.88	F = 0.261 Sig = 0.612
	Stand_to_sit (s)	−0.60 ± 1.77	−0.31 ± 2.43	F = 0.428 Sig = 0.516	0.32 ± 1.37	−2.15 ± 2.54	F = 0.519 Sig = 0.475
	TOTAL (s)	−1.06 ± 4.23	−3.03 ± 4.62	F = 0.753 Sig = 0.390	−0.17 ± 5.62	1.56 ± 5.42	F = 0.203 Sig = 0.655

** *p* < 0.001; Mean ± Sd; CG: Control group; EC: Experimental group; FTSTS: Five times sit to stand test; HGS: Handgrip strength; Kg: Kilograms; TUG: Timed Up and Go test; s: Seconds.

**Table 4:** Quality of life scores (intergroup differences).

		Pre		Post	
		EG (*n* = 13)	CG (*n* = 11)	EG (*n* = 13)	CG (*n* = 11)
SF-12	Physical Score	48.81 ± 9.45	42.06 ± 12.71	48.52 ± 9.01	44.41 ± 9.88
	Mental Score	48.81 ± 8.70	50.39 ± 10.47	53.41 ± 8.65	55.16 ± 9.53

CG: Control group; EC: Experimental group; SF-12: 12-Item Short Form Survey.

**Table 5:** Quality of life scores (intragroup and multivariant differences).

		Differences between Moments (Post-Pre)		
		EG (*n* = 13)	CG (*n* = 11)	Manova 2 × 2
		Dif. Post-Pre	Dif. Post-Pre	
SF-12	Physical Score	−0.29 ± 6.26	2.34 ± 13.09	F = 2.677 Sig = 0.019
	Mental Score	4.60 ± 8.38	4.76 ± 13.95	F = 0.114 Sig = 0.738

CG: Control group; EC: Experimental group; SF-12: 12-Item Short Form Survey.

**Table 6:** Sickness, usability, and post gaming experience in the experimental group.

		EG (*n* = 13)	
		Intermediate	Post
Simulator Sickness Questionnaire		No symptoms	No symptoms
System Usability Scale		76.73 ± 10.77	73.96 ± 16.77
Game Experience Questionnaire (post game module)	**PE (0–4)**	2.77 ± 0.68	2.87 ± 0.86
	**NE (0–4)**	0.15 ± 0.29	0.22 ± 0.21
	**T (0–4)**	0.35 ± 0.37	0.37 ± 0.43
	**RR (0–4)**	0.33 ± 0.45	0.19 ± 0.33

0–4: Not at all to Extremely; EG: Experimental group; PE: Positive experience; NE: Negative experience; RR: Returning to reality; T: Tiredness.

**Table 7:** Ad hoc satisfaction questionnaire and responses from participants.

		Experimental Group (*n* = 13)	
		*n*	%
How did you find the experience?	Good or very good	13	100%
What did you like the most?	Everything	6	54.54%
	The exercise I did without realizing	2	18.18%
	What I saw in the virtual world	2	18.18%
	Nothing in particular	1	9.09%
	Following a routine	1	9.09%
	Completing the program	1	9.09%
Was there anything you did not like?	No	10	72.73%
	Initial fear due to not knowing what to expect	2	18.18%
	Difficulties in use	1	9.09%
Would you recommend the IVR experience?	Yes	11	81.82%
	No	2	18.18%
Do you think this could be useful for people of your age? Why?	Yes “You exercise your mental agility and many other things that we need” ”It is energizing” “It is fundamental to remain active” “To do exercise” “It´s important for people of my age to move” “You get more agile, amongst other benefits” “You learn new things” “It encourages you to do exercise” “It helps you see and feel things that you would not feel on your own” “It is a good way to exercise, even if it is a little difficult at first”	11	81.82%
	No “I don´t find it interesting” “I don´t find it useful”	2	18.18%

## Data Availability

The data presented in this study are available on request from the corresponding author.
